# Refractory Chronic Spontaneous Urticaria: A Rare Presentation of Graves' Disease

**DOI:** 10.7759/cureus.112044

**Published:** 2026-07-04

**Authors:** Maryam Qaiser, Nazil Anwar, Suham Amin

**Affiliations:** 1 Acute Medicine, Stepping Hill Hospital, Stockport NHS Foundation Trust, Stockport, GBR

**Keywords:** chronic urticaria, cutaneous graves disease, graves disease, hyperthyroidism, periorbital angioedema, persistent angioedema, recurrent widespread urticaria, skin manifestation of graves disease, thyroid autoimmunity, wheals

## Abstract

Chronic spontaneous urticaria (CSU) is marked by repeated episodes of wheals and/or angioedema lasting more than six weeks and occasionally coexists with autoimmune thyroid disease. Autoimmune thyroid disease may contribute to CSU in a subset of patients, and symptoms can be refractory to conventional antihistamine and corticosteroid therapy. A 33-year-old woman with a history of asthma presented with recurrent widespread urticaria and periorbital angioedema, often triggered by food, resulting in repeated acute care visits. Symptoms were minimally responsive to high-dose antihistamines and systemic corticosteroids. She underwent evaluation for mast cell disorders and neuroendocrine tumors, which were excluded through normal serum tryptase, urinary 5-hydroxyindoleacetic acid, and plasma metanephrine levels. Biochemical testing revealed suppressed thyroid-stimulating hormone (TSH) and elevated free thyroid hormones, with positive TSH receptor antibodies, consistent with autoimmune thyroid disease. Medical management with antithyroid therapy, beta-blockers, and antihistamines achieved biochemical control of hyperthyroidism but did not improve urticaria. Thyroid ultrasound demonstrated diffuse hypervascular enlargement. Given persistent severe CSU and refractory hyperthyroidism, the patient underwent total thyroidectomy. Histopathological examination confirmed autoimmune thyroid disease. Following surgery, urticaria and angioedema resolved completely, with no recurrence at follow-up. This case illustrates a severe autoimmune CSU phenotype likely mediated by overlapping IgE- and IgG-driven mechanisms sustained by active thyroid autoimmunity. The complete resolution of urticaria following thyroidectomy suggests a direct causal relationship. Early recognition of thyroid dysfunction in patients with recurrent or refractory CSU can guide targeted management, prevent repeated hospital visits, and, in select cases, may enable definitive thyroid ablation as a curative approach. Multidisciplinary evaluation and individualized treatment planning are essential for patients presenting with severe autoimmune CSU unresponsive to conventional therapy.

## Introduction

Chronic urticaria is defined as the recurrent occurrence of itchy wheals (hives), angioedema, or both, lasting for more than six weeks. When no specific external trigger is identified, it is classified as chronic spontaneous urticaria (CSU) [1]. A significant subset of CSU (30-45%) is considered to have an autoimmune basis, often associated with functional autoantibodies [2,3]. This autoimmune association is further supported by its frequent comorbidity with other autoimmune diseases, including autoimmune thyroid disease (ATD) [1,4,5].

The pathogenesis of autoimmune CSU is understood through two main endotypes. Type I CSU involves an "autoallergic" IgE-mediated pathophysiology, where IgE autoantibodies (e.g., anti-thyroid peroxidase (anti-TPO)) can trigger mast cell degranulation [1,6]. In contrast, Type IIb CSU is a classic autoimmune form driven by IgG autoantibodies targeting the high-affinity IgE receptor (FcεRIα) or IgE itself, leading to direct activation of basophils and mast cells. Clinically, these endotypes are not mutually exclusive and often overlap (Type I/IIb) in the same patient, reflecting a complex autoimmune landscape [4]. 

We present a case that is significant because it demonstrates complete resolution of CSU after total thyroidectomy, suggesting a potential association between thyroid disease and CSU in selected patients. 

## Case presentation

**Figure 1:**
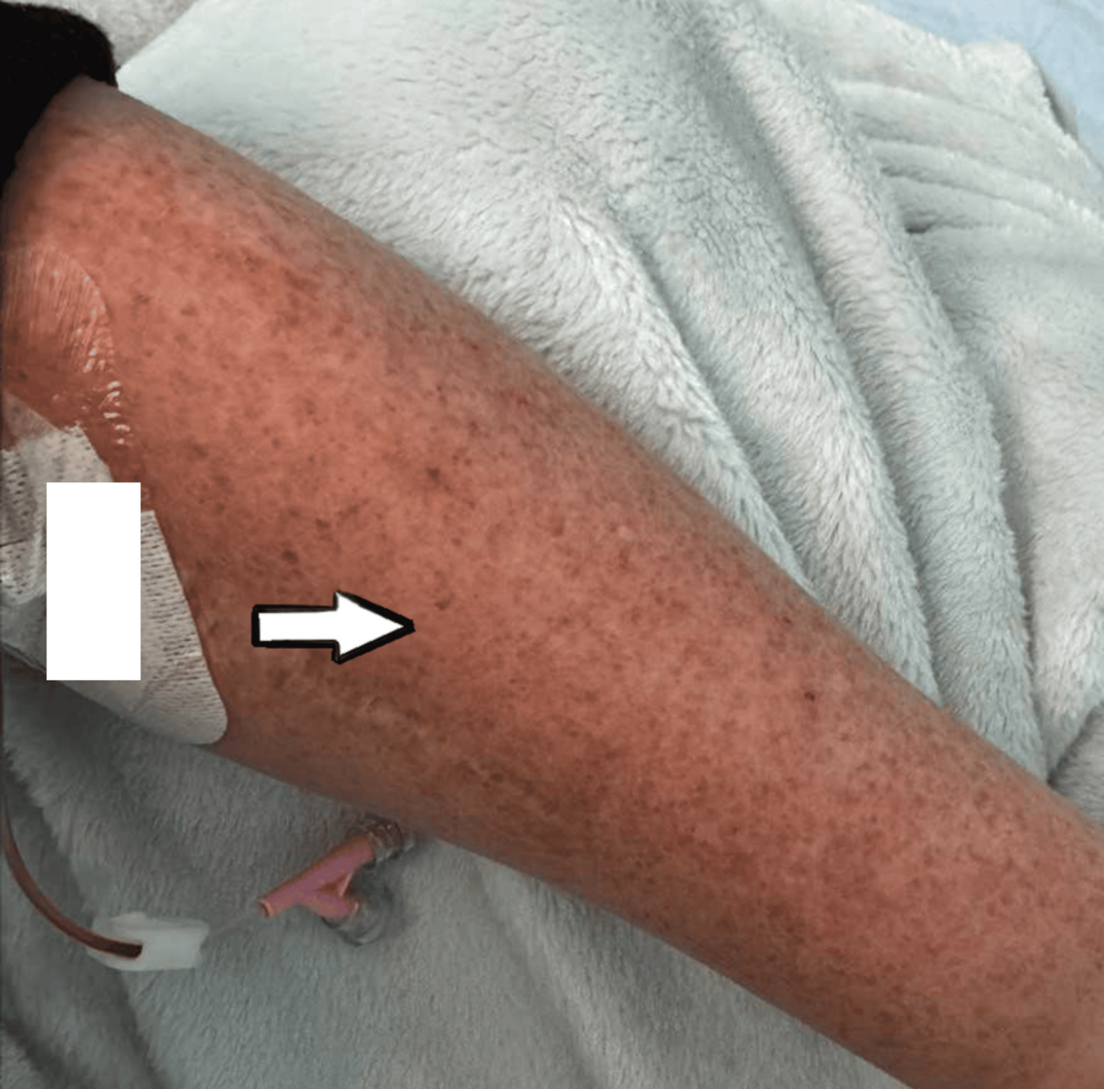
Acute episode of chronic urticaria on arms

**Figure 2:**
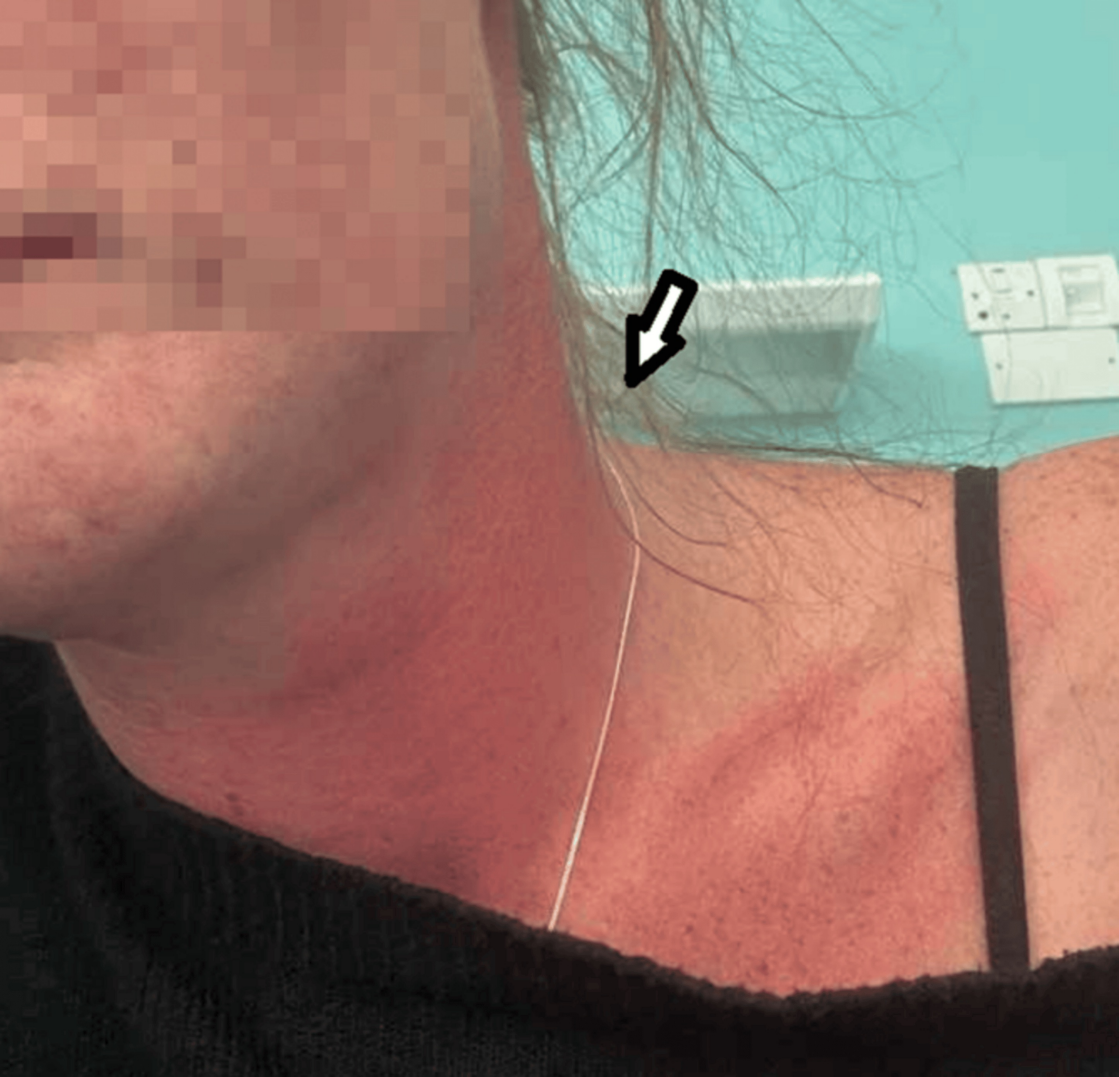
Flare of chronic urticaria following beverage intake

A 33-year-old female patient with a history of asthma presented with recurrent episodes of widespread erythematous urticaria affecting the face, neck, arms, and torso, accompanied by periorbital angioedema (Figures 1, 2). The episodes showed a temporal association with the ingestion of coffee with cinnamon and, later, tea.

Initial management with systemic corticosteroids and antihistamines provided minimal relief. Baseline investigations were normal, as shown in Table 1. Concurrent evaluation revealed biochemical thyrotoxicosis (suppressed TSH, elevated free T4 (FT4), and free T3 (FT3)) and elevated TSH receptor antibodies (13 IU/L; normal <0.9) (Table 2), leading to a diagnosis of Graves' disease.

**Table 1:** Laboratory investigations eGFR: estimated glomerular filtration rate

Test	Patient Value	Reference Range
White Blood Cells	10.4	3.7–11.0 ×10⁹/L
Hemoglobin	11.4	Male: 13.5–17.5 g/dL; Female: 12.0–15.5 g/dL
Mean Corpuscular Volume	79.2	80–100 fL
Platelets	287	150–450 ×10⁹/L
Neutrophils	6.7	1.7–7.5 ×10⁹/L
Lymphocytes	2.5	1.0–4.0 ×10⁹/L
Sodium (Na⁺)	144	133–146 mmol/L
Potassium (K⁺)	4.6	3.5–5.3 mmol/L
Urea	4.1	2.5–7.8 mmol/L
eGFR	>90	≥90 mL/min/1.73 m²

**Table 2:** Specialty investigations TSH: thyroid-stimulating hormone; HIAA: hydroxyindoleacetic acid

Test	Patient Value	Reference Range
Serum 5-HIAA	39	0.1—140 nmol/L
Serum Tryptase	1.9	<12.9 µg/L
TSH	<0.0.1	0.4–4.0 mIU/L
Serum Free T3	25.7	3.1–6.8 pmol/L
Serum Free T4	54.5	12–22 pmol/L
TSH Receptor Antibodies	13	<1.75 IU/L

Treatment was initiated with carbimazole and subsequently switched to propylthiouracil (PTU), alongside beta-blockade and ongoing antihistamines. This regimen controlled the hyperthyroidism but did not resolve the urticarial flares. The urticaria persisted despite high-dose antithyroid therapy (PTU 600 mg daily) and varying doses of oral prednisolone.

**Figure 3:**
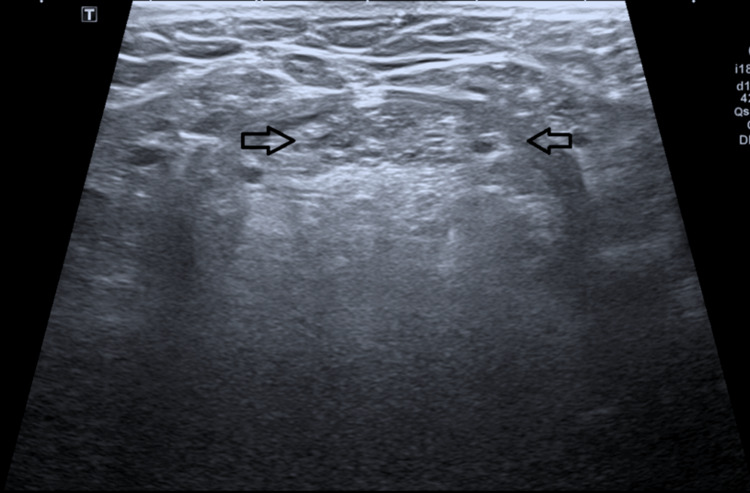
Ultrasound of the thyroid demonstrating mild enlargement, demonstrating inhomogeneous echo pattern with pseudonodular appearance/micronodules and mild increase in vascularity, with features suggestive of chronic autoimmune thyroiditis.

Further investigations ruled out other causes of flushing and anaphylactoid reactions, with normal serum tryptase, 24-hour urine 5-hydroxyindoleacetic acid (5-HIAA), and plasma metanephrines. Thyroid ultrasound confirmed a mildly enlarged thyroid gland with a heterogeneous echotexture, a pseudonodular appearance characterized by micronodules, and mildly increased vascularity (Figure 3).

Omalizumab was not offered, as the patient preferred a definitive treatment due to the significant impact of her symptoms on daily functioning. Radioactive iodine was offered but declined because of concerns related to her work in a clinical setting. Due to refractory thyrotoxicosis and persistent, debilitating urticaria, which was suspected to be related to her autoimmune thyroid state, a total thyroidectomy was performed. Histopathological examination confirmed benign follicular hyperplasia with marked lymphocytic infiltration, diagnostic of Graves' disease. Following thyroidectomy and the initiation of levothyroxine replacement therapy, the patient's urticaria and angioedema resolved completely by the six-month follow-up, which shows a potential association between thyroid disease and CSU in selected patients.

## Discussion

The association between CSU and ATD is well-documented, with a significant portion of CSU patients exhibiting thyroid autoantibodies or dysfunction [5]. Cases of CSU associated specifically with Graves' disease have been reported, often showing improvement of urticaria with antithyroid treatment [7,8]. A few such cases are given in Table 3. 

**Table 3:** Studies showing significant improvement or resolution in CSU after thyroid therapy CSU: chronic spontaneous urticaria

Study	CSU Outcome
Agarwal & Faas (2014) [9]	Complete resolution after euthyroid state
Nasir & Tahir (2019) [10]	Improvement with thyroid control
Košec et al. (2020) [11]	Complete resolution after thyroidectomy

The present case is notable for its severity and refractoriness. Unlike previous reports where urticaria responded to carbimazole [8] or levothyroxine in patients with TPO antibodies [12], this patient's symptoms persisted despite high-dose propylthiouracil and systemic corticosteroids, requiring total thyroidectomy for definitive resolution. This suggests a phenotype where CSU is not merely a parallel autoimmune comorbidity but is intrinsically driven by the presence of the autoimmune thyroid tissue itself, representing a severe end of the clinical spectrum.

The pathophysiology in this case likely involves an overlap of the recognized autoimmune endotypes. The clinical presentation, including trigger-associated flares and a history of asthma, suggests a Type I (autoallergic) endotype, potentially mediated by IgE autoantibodies against self-antigens such as thyroid peroxidase (TPO) [6]. Concurrently, the presence of active Graves' disease indicates a robust systemic autoimmune state that can drive a Type IIb endotype, characterized by functional IgG autoantibodies against FcεRIα or IgE itself [6,13]. This overlap is common, with one study noting it in 51% of CSU patients [1]. Mendelian randomization studies support a causal role for Graves' disease in urticaria, potentially mediated through alterations in specific immune cell populations like T cells [14]. In this case, the thyroid gland may have acted as a persistent source of immune stimulation or antigen, sustaining mast cell and basophil activation in the skin until it was removed.

For patients with CSU refractory to first-line antihistamines and corticosteroids, especially when associated with active Graves' disease, alternative management strategies must be considered. Biologic therapy, such as omalizumab (anti-IgE), is a targeted and effective therapy for refractory CSU. It may be particularly beneficial in patients with a suspected Type I (IgE-mediated) component, as it neutralizes circulating IgE, including pathogenic IgE autoantibodies [8]. Immunosuppressive agents such as cyclosporine can be effective for severe autoimmune CSU by inhibiting T-cell activation and cytokine production, though their use is limited by potential toxicity [4,6]. When CSU is refractory and closely linked to Graves' disease, total thyroidectomy or radioactive iodine (RAI) ablation should be regarded as a definitive therapeutic option. This approach addresses the underlying autoimmune focus, potentially curing both the hyperthyroidism and the associated urticaria [14].

Early and comprehensive diagnostics are crucial to identify such patients promptly, prevent protracted morbidity, and guide appropriate therapy. For all patients with CSU, initial screening should include a full Thyroid Panel, including serum TSH and free T4, to assess thyroid function [15]. Thyroid autoantibodies, such as anti-TPO antibodies as a marker of general thyroid autoimmunity, and thyrotropin receptor antibodies (TRAb) for the specific diagnosis of Graves' disease, should be considered [8,14]. Autologous serum skin test (ASST) is a simple screening tool for autoimmune (Type IIb) CSU. A positive ASST can identify patients more likely to have an autoimmune basis and associated conditions like ATD [4].

Consistent with our findings, Ruggeri et al. reported a series of patients with chronic spontaneous urticaria associated with ATD. These findings support a potential relationship between thyroid autoimmunity and CSU and suggest that thyroidectomy may result in symptom remission in selected patients [16].

## Conclusions

This case exemplifies a severe, treatment-resistant form of CSU directly linked to Graves' disease, consistent with an overlapping Type I/IIb autoimmune endotype. It underscores the critical importance of comprehensive thyroid screening, including functional tests and autoantibodies, in all patients presenting with CSU. Furthermore, it highlights that in refractory cases, treatment should consider going beyond symptomatic control to include targeted biologics or, ultimately, definitive management of the underlying Graves' disease. Early diagnosis through systematic evaluation can facilitate timely and potentially curative interventions, significantly improving patients' quality of life.
